# Preliminary Investigation into the Predation of *Pomacea canaliculata* by *Aquatica leii* Larvae

**DOI:** 10.3390/insects17030297

**Published:** 2026-03-09

**Authors:** Jiangtao Luo, Chunlin An, Yingjun Wu, Huachao Xu

**Affiliations:** 1 College of Forestry and Biotechnology, Zhejiang Agricultural and Forestry University, Hangzhou 311300, China; a1254692245@163.com (J.L.); lindeninsects@yeah.net (C.A.); 2 Suichang County Ecological Forestry Development Center, Lishui 323000, China; scxwyj@163.com

**Keywords:** *Aquatica leii*, *Pomacea canaliculata*, biological control, instar difference, midgut extract

## Abstract

*Pomacea canaliculata*, a pervasive invasive gastropod, inflicts significant ecological and economic damage in Chinese rice ecosystems. With the limitations of chemical molluscicides, sustainable biological control solutions are urgently required. This study presents a comprehensive investigation into the biocontrol potential of larvae of the endemic aquatic firefly, *Aquatica leii*, against *Pomacea canaliculata*. Through controlled laboratory experiments, we evaluated the feeding preference of larvae when offered a choice between *Pomacea canaliculata* and a native snail (*Cipangopaludina chinensis*), and systematically quantified the predatory efficiency (lethal time and consumption amount) across the 3rd to 6th larval instars. Furthermore, the lethal activity of crude extracts from distinct anatomical regions of the larval digestive tract (mouthpart, foregut, midgut, and hindgut) was assayed via injection into *Pomacea canaliculata*. The larvae accepted *Pomacea canaliculata* as a viable prey source. Predatory performance varied markedly among instars; 4th-instar larvae exhibited optimal efficacy, characterized by the shortest mean lethal time (7.37 min) and the highest mean consumption (1.23 g). Midgut extract was identified as the principal causative agent of mortality, inducing a 96.7% mortality rate in *Pomacea canaliculata*, which was significantly superior to the minimal effects observed from other extract types. This points to the midgut secretion as a likely source of potent bioactive compounds responsible for rapid snail lethality, warranting further investigation. responsible for rapid snail lethality. Our results conclusively demonstrate, from both behavioral and physiological vantage points, the feasibility of *Aquatica leii* larvae as a highly effective native biocontrol agent. This work establishes a critical foundation for future research aimed at the isolation and characterization of the midgut-specific active substances, paving the way for the development of novel, target-selective biogenic molluscicides.

## Simple Summary

The search for environmentally friendly ways to control the invasive *Pomacea canaliculata* has led scientists to look at its natural predators. This study focuses on the larvae of *Aquatica leii*, a freshwater firefly native to China. We wanted to see if these underwater larvae could effectively prey on the destructive *Pomacea canaliculata*. In our lab tests, not only did the firefly larvae successfully attack and eat the *Pomacea canaliculata*, but we also found that one particular growth stage—the 4th-instar larvae—was the most effective hunter, killing *Pomacea canaliculata* the fastest and consuming the most. A particularly notable finding was that liquid extracted specifically from the larval midgut exhibited strong lethal activity against *P. canaliculata* under experimental conditions, causing over 96% mortality. This suggests that the larvae may use potent injected toxins to subdue their prey, although further verification is needed. against the *Pomacea canaliculata*, causing over 96% mortality. This shows that the larvae likely use a powerful injected toxin to subdue their prey. Our research highlights *Aquatica leii* as a promising, natural candidate for biocontrol and suggests that its unique midgut toxin could be the key to developing new, targeted *Pomacea canaliculata* control products that are safer for the environment.

## 1. Introduction

*Pomacea canaliculata* (Gastropoda: Mesogastropoda: Ampullariidae) [1] is a widespread South American freshwater mollusk whose range is rapidly expanding and is now becoming a common component of the fauna of many countries in Southeast Asia. It is listed among China’s first cohort of invasive alien species. Listed among China’s first cohort of invasive alien species, it has rapidly spread since its introduction, colonizing paddy fields, ditches, and wetland ecosystems across multiple southern provinces [2]. This snail exhibits a broad diet and high reproductive capacity, causing severe damage to economically important aquatic crops such as rice, water bamboo, and lotus root. This leads to significant yield losses, quality degradation, and substantial annual economic damage [3]. Statistics indicate that the infestation area of *P. canaliculata* in China had reached 1.7011 million hectares by 2020 [4].

Current control strategies for *P. canaliculata* are generally categorized into physical and chemical methods. Chemical control relies heavily on molluscicides such as niclosamide ethanolamine salt (NES) and metaldehyde (MD) [5]. However, their long-term and extensive use raises concerns regarding pesticide residues, water pollution, and potential threats to aquaculture and ecological security. Physical methods, such as manual collection of snails and egg masses, are notably inefficient [6]. Consequently, developing environmentally friendly and sustainable biological control technologies has become an urgent need for managing *P. canaliculata*.

Some practical attempts have been made at biological control using aquatic animals like ducks and soft-shelled turtles to consume *P. canaliculata* [7,8]. However, large-scale application is often limited by farming conditions, control precision, and scope of applicability. Finding a natural enemy organism that is adapted to aquatic environments, highly specific, and offers efficient control presents a new direction for managing this invasive snail.

Fireflies (Insecta: Coleoptera: Lampyridae) [9,10,11], represent an important group of resource insects. The larvae of many terrestrial species feed on mollusks such as snails and slugs, demonstrating considerable potential for the biological control of agricultural and forestry pests [12,13]. *Aquatica leii* is an aquatic firefly species endemic to China, belonging to the family Lampyridae [14]. Its entire larval stage occurs in clean aquatic environments such as streams and paddy fields, where it preys on small snails and bivalves [15]. Therefore, this study considers it to possess the biological basis for acting as a potential natural enemy against *P. canaliculata*.

To date, research on the biological characteristics and ecological functions of *A. leii*, both domestically and internationally, remains in its early stages [16,17,18]. There is a lack of systematic reports on its predatory capability against *P. canaliculata*, its feeding preferences, or its control efficacy. Therefore, this study employs *A. leii* larvae as test subjects. By measuring parameters such as lethal time and consumption rate per unit time against *P. canaliculata*, we preliminarily assess the potential of *A. leii* for application in the biological control of *P. canaliculata*. The aim is to provide a scientific basis for the eco-friendly management of *P. canaliculata* and the utilization of aquatic fireflies as a biological resource.

## 2. Materials and Methods

### 2.1. Experimental Materials

#### 2.1.1. Test Insects

The larvae of *A. leii* used in this experiment were all purchased in 2024 from firefly breeding bases in Jiangxi and Fujian provinces, China. To ensure physiological uniformity, only larvae originating from the same captive-reared population (maintained under standardized conditions for multiple generations) were used. All larvae were laboratory-reared and supplied with detailed rearing history, including diet (freshwater snails) and environmental conditions (28 ± 1 °C, 14 L:10 D photoperiod). *A. leii* undergoes complete metamorphosis, with a life cycle consisting of egg, larval (typically 6 instars), pupal, and adult stages. The larval stage lasts approximately 6–8 months under natural conditions, during which larvae molt 5–6 times before pupation [15,16]. Instar determination was based on head capsule width measurement and molting records, following the criteria established by Fu et al. [16]. Only larvae of the same instar and similar body size were selected for experiments.

#### 2.1.2. Prey

*P. canaliculata* exhibits an extremely high reproductive rate, with juvenile snails reaching sexual maturity within just three months of development [19]. Consequently, the period before sexual maturity represents the optimal window for its control. Therefore, in this experiment, *P. canaliculata* individuals weighing 0.5–1 g (shell height approximately 10–15 mm) were used as the target prey, while *Cipangopaludina chinensis* (Gastropoda: Architaenioglossa: Viviparidae), a native sympatric snail species, served as the reference prey (weighing 0.8–1.2 g, shell height approximately 15–20 mm). All snails were collected from multiple irrigation ditches and ponds within a 5 km radius of Hangzhou Xiaoshan International Airport (30.23° N, 120.26° E), Zhejiang Province, China. To ensure genetic diversity and representativeness, snails were pooled from at least five distinct collection sites. Immediately after collection, snails were subjected to a 7-day quarantine period in the laboratory under controlled conditions (28 ± 1 °C, aerated dechlorinated water, natural light cycle). During quarantine, snails were visually inspected for physical damage, abnormal behavior, and the presence of parasites or egg masses. Only apparently healthy, active snails with intact shells were used. To acclimate snails to laboratory conditions and minimize stress, they were held in aquaria with dechlorinated water and fed fresh lettuce ad libitum for at least 3 days prior to experiments.

*C. chinensis* was chosen as the reference prey because it is a native sympatric snail species commonly found in the same habitats as *P. canaliculata* in southern China, and it represents a natural prey item for *A. leii* larvae [18]. This allows assessment of prey choice in a context relevant to the firefly’s natural foraging behavior.

### 2.2. Experimental Methods

#### 2.2.1. Larval Pre-Treatment

Prior to experiments, all healthy and active *A. leii* larvae (3rd to 6th instar) were subjected to a standardized 7-day starvation period in a constant temperature incubator (Ningbo Jiangnan Instrument Factory, RXZ-328A, Ningbo, China) under a fixed photoperiod (L:D = 14 h:10 h) to normalize their hunger level.

#### 2.2.2. Feeding Preference Assay

Randomly selected, pre-treated larvae (3rd to 6th instar, equal numbers per instar) were used as test insects and divided into 4 groups by instar, with 15 individuals per group. The assay was conducted in 15 cm diameter Petri dishes (Nest Biotechnology, 150101, Wuxi, China) lined with moist filter paper. The central area was temporarily divided into three equal-sized (sector-shaped) compartments by removable partitions. A single starved larva was placed in the central compartment. After a 5 min acclimation period, the partitions were removed, and one live *P. canaliculata* and one live *C. chinensis* of similar weight (approx. 1.0 g) were simultaneously placed in the two opposing (sector-shaped) compartments. A total of 36 independent trials were conducted using new larvae and snail individuals for each trial. Larvae were randomly selected from the 3rd to 6th instar groups (15 individuals per instar) to ensure representation across all instars. Larval behavior was recorded via video during a 2 h observation period. Recorded metrics included the first attack target (defined as contact with the mouthparts and attempted predation) and the final feeding target (sustained feeding for over 5 min). This experiment aimed to determine the fundamental feeding preference of *A. leii* larvae.

#### 2.2.3. Determination of Lethal Time of *Pomacea canaliculata* by *Aquatica leii* Larvae at Different Instars

For each larval instar, 15 pre-treated larvae were randomly divided into three subgroups (*n* = 5 per subgroup), which served as three biological replicates. Thus, “three replicate treatment groups” refers to these three parallel subgroups run simultaneously. The experiment was performed once for each instar, with each larva tested individually. Mortality of *P. canaliculata* was confirmed when the snail showed no movement, failed to respond to gentle probing of the foot with a blunt needle, and retracted deeply into the shell for more than 5 min. In cases of uncertainty, observation was extended for an additional 10 min. Control groups (snails without larvae) were maintained under identical conditions to monitor background mortality.

#### 2.2.4. Weekly Consumption Amount of *Pomacea canaliculata* by *Aquatica leii* Larvae at Different Instars

For each larval instar, 15 pre-treated larvae were randomly divided into three subgroups (*n* = 5 per subgroup) as three biological replicates. This experiment was conducted independently from the lethal time assay, using a new set of larvae and snail tissue. Each larva was provided with 5 g of fresh *P. canaliculata* tissue (dissected from live snails, excluding the shell). To correct for autogenic changes in tissue weight (e.g., water loss), three control containers without larvae were set up in parallel. The amount consumed per larva over 7 days was calculated as: (initial tissue weight—remaining tissue weight)—(mean weight loss in control containers). Tissue weight was measured after gently blotting with absorbent paper.

#### 2.2.5. Determination of Lethal Efficiency of *Aquatica leii* Digestive Tract Fluids against *Pomacea canaliculata*

Sixty healthy 4th-instar *A. leii* larvae were immobilized by brief exposure to −20 °C for 1 min in a cryogenic freezer (Haier, DW-40L508, Qingdao, China). Dissection was performed on a wax plate under a stereomicroscope (Nanjing Jiangnan Novel Optics, JSZ6, Nanjing, China) in chilled physiological saline. The entire digestive tract was excised and divided into four anatomical regions based on external features: buccal region (mouthparts and pharynx), foregut (esophagus and crop), midgut (anterior and posterior sections, distinguished by darker pigmentation and presence of food bolus), and hindgut (intestine and rectum). Each section was weighed and homogenized manually in ice-cold physiological saline at a ratio of 1:1 (*w*/*v*). The homogenate was centrifuged at 4 °C, 6000 rpm for 15 min (Eppendorf, 5424R, Hamburg, Germany), and the supernatant was collected. The final concentration of the supernatant corresponded to approximately 0.5 g of tissue per mL (original tissue diluted 1:1 with saline).

Prior to the main experiment, a dose-finding pre-test was conducted using midgut extract at doses of 5, 10, and 20 µL per snail (*n* = 5 per dose). The 10 µL dose was selected because it induced clear mortality without causing immediate death from injection trauma, allowing assessment of extract-specific effects.

For the bioassay, 10 µL of each supernatant was injected into the foot muscle of individual *P. canaliculata* (0.5–1.0 g) using a 50 µL Hamilton syringe (Hamilton, 80965, Reno, NV, USA) fitted with a 30-gauge (30 G) needle (KDL, 30 G, Shanghai, China). Four treatment groups (buccal, foregut, midgut, hindgut extracts) and three control groups were included: (1) physiological saline (vehicle control), (2) sterile water (to control for osmotic effects), and (3) sham injection (needle insertion without fluid injection) to account for physical injury. Each group comprised 30 snails, divided into three replicates of 10 snails each. Mortality was recorded at 1, 3, 6, and 12 h post-injection; snails were considered dead if they showed no movement and failed to respond to gentle prodding.

#### 2.2.6. Data Analysis

Data were analyzed using SPSS Statistics version 26.0 (IBM Corp., Armonk, NY, USA). For the feeding preference assay, a chi-square goodness-of-fit test [20] was used to compare observed frequencies of first attack and final feeding choices against an expected 1:1 distribution. For lethal time and consumption amount, data were first tested for normality (Shapiro–Wilk test) and homogeneity of variances (Levene’s test). As all data met these assumptions (*p* > 0.05), one-way analysis of variance (ANOVA) was performed to compare means among instars, followed by Tukey’s honestly significant difference (HSD) post hoc test for multiple comparisons. For the extract injection experiment, mortality rates among treatment groups were compared using Fisher’s exact test (due to small expected frequencies in some cells). A significance level of significance level (α) of 0.05 was used for all tests.

## 3. Results

### 3.1. Feeding Preference

To determine whether the larvae of *A. leii* actively select *P. canaliculata* as prey and to evaluate their choice preference in the presence of different prey items—thereby assessing their targeted predation potential against *P. canaliculata* in natural environments—a two-choice experiment was conducted, with the results summarized in Table 1.

The chi-square goodness-of-fit test results indicated that while the larvae of *A. leii* showed a tendency to preferentially select *C. chinensis* as the initial attack target, this difference was not statistically significant (χ^2^ = 2.78, *p* > 0.05). Similarly, no significant difference was observed in the proportion of larvae that ultimately fed on either snail species (χ^2^ = 1.00, *p* > 0.05). These results suggest that *A. leii* larvae are capable of and willing to accept *P. canaliculata* as a food source. However, it should be noted that the total number of replicates (36 across all instars combined) may limit the statistical power of the chi-square test to detect small but biologically meaningful preferences. Furthermore, prey choice experiments were conducted only at a 1:1 ratio, which does not capture potential density-dependent shifts in feeding decisions that may occur in natural habitats where prey abundance varies. Future studies should incorporate multiple prey density ratios to better understand the foraging behavior of *A. leii* under ecologically relevant conditions.

### 3.2. Lethal Time

**Figure 1:**
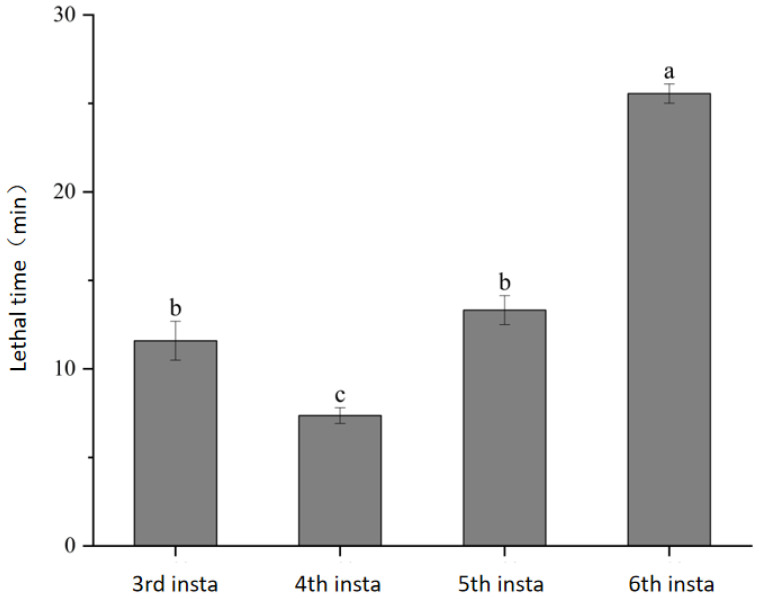
Lethal Time of *Aquatica leii* Larvae at Different Instars Against *Pomacea canaliculata*. Means (±standard error, SE) followed by the same letters in the column do not differ significantly by Tukey’s test (*p* > 0.05). Note: X-axis labels “3rd”, “4th”, “5th”, and “6th” refer to the corresponding larval instars.

This study found that the first and second instar larvae of *A. leii* were incapable of preying on intact *P canaliculata*, only consuming processed snail tissue. This observation is consistent with findings reported by Guo Zhaoxiang in a study on *Pyrocoelia pectoralis* [21]. To accurately assess the actual predatory effect of each larval instar, these two early instars were excluded from the experimental design. To facilitate observation and eliminate interference from factors such as natural mortality of *P. canaliculata*, individual snails were housed separately in transparent rearing containers measuring 4.0 cm × 4.0 cm × 2.5 cm. Under constant temperature conditions of 28 °C, the lethal time exerted by third, fourth, fifth, and sixth instar larvae on *P. canaliculata* was determined (Figure 1).

The data indicate that the lethal time required by sixth-instar larvae to kill *P. canaliculata* was significantly longer than that of all other instars, with a mean of 25.89 min, suggesting the slowest predatory process against *P. canaliculata* at this stage. In contrast, fourth-instar larvae exhibited the shortest mean lethal time of only 7.37 min, which differed significantly from the third-, fifth-, and sixth-instar groups, indicating that fourth-instar larvae likely possess the strongest lethal capacity against *P. canaliculata*.

The lethal times of third- and fifth-instar larvae were relatively similar, at 11.26 min and 12.99 min respectively, with no statistically significant difference between them. However, both were significantly longer than that of the fourth-instar group and shorter than that of the sixth-instar group. These results demonstrate that lethal time does not simply decrease or increase progressively with larval instar.

### 3.3. Measurement of Consumption Amount

**Figure 2:**
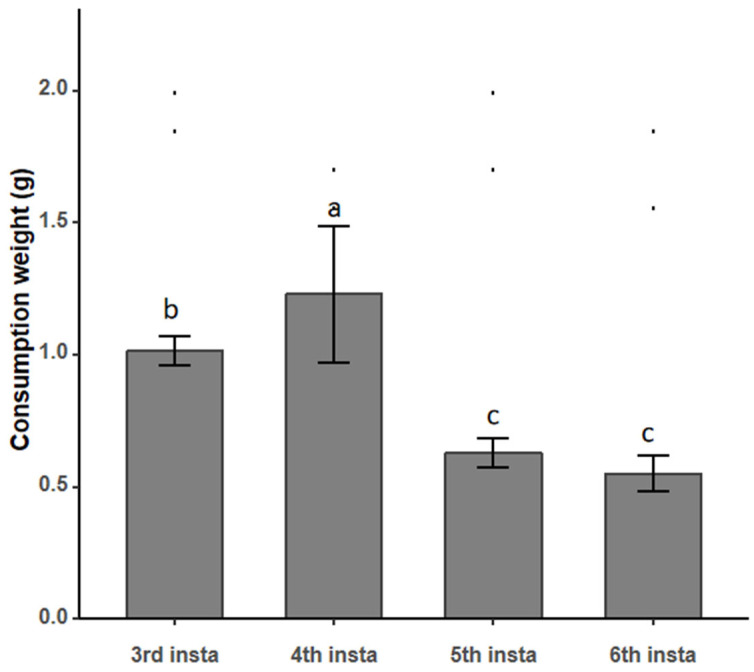
Weekly Consumption Amount of *Pomacea canaliculata* by *Aquatica leii* Larvae at Different Instars. Means (±SE) followed by the same letters in the column do not differ significantly by Tukey’s test (*p* > 0.05). Black dots represent individual data points or outliers for each instar group, showing the distribution of raw measurements. Note: x-axis labels “3rd”, “4th”, “5th”, and “6th” refer to the corresponding larval instars.

Prey consumption is a key metric for evaluating the predatory efficacy and energy acquisition strategy of a natural enemy. To systematically analyze the nutritional niche and utilization efficiency of *A. leii* larvae at different instars toward their prey, this study, following the recording of lethal time, measured the actual amount of *P. canaliculata* tissue consumed per unit time by larvae of each instar (with three replicates per instar) (Figure 2).

As shown in Figure 2, the average consumption amounts of larvae at different instars exhibited significant inter-group differences. Among them, the fourth-instar larvae showed the highest consumption, with an average of 1.23 g, whereas the sixth-instar larvae showed the lowest consumption, averaging only 0.55 g. Overall, larvae at the third and fourth instars had relatively higher consumption levels, while consumption tended to decrease as larval development progressed beyond these stages.

### 3.4. Lethal Efficiency of Aquatica leii Digestive Tract Fluids Against Pomacea canaliculata

This study has confirmed that *A. leii* larvae are capable of preying upon *P. canaliculata* and accept it as a food source. The feeding process of lampyrid larvae is not merely a matter of physical ingestion. Studies have shown that they primarily inject digestive tract fluids rich in various enzymes (such as proteases and lipases) into their prey [13] to facilitate external pre-digestion, thereby liquefying and absorbing the prey’s tissues [18]. Consequently, digestive tract fluid is a key physiological factor enabling firefly larvae to kill and digest mollusk prey [13]. Therefore, this study investigated the direct effect of in vitro digestive tract fluids from *A. leii* on *P. canaliculata* (Table 2) to further elucidate its potential as a biocontrol agent.

The data presented in Table 2 clearly demonstrate that the midgut extract exhibited exceptionally strong lethal activity, causing the death of 27, 30, and 30 *P. canaliculata* individuals across the three replicate groups, respectively, resulting in an overall mortality rate as high as 96.7%. In contrast, the lethal effects observed in the other treatment groups were minimal.

## 4. Discussion

The strong reproductive capacity of *P. canaliculata* and the lack of effective natural enemies to control it make the management of this invasive species particularly challenging [22]. This study found that the larvae of the aquatic firefly *A. leii* possess clear predatory capability and lethal potential against *P. canaliculata*. The results indicate that although *A. leii* larvae show a preference for the native snail *Cipangopaludina chinensis* under free-choice conditions, they also exhibit a positive willingness to feed on *P. canaliculata*. This finding holds significant ecological importance: in habitats where *P. canaliculata* has invaded extensively and displaced native snail species, *A. leii* can utilize the invader as an effective alternative prey resource. This allows the firefly population to persist and continue exerting pest control functions. This ability reduces the dependency of its control efficacy on specific native prey, thereby enhancing its environmental adaptability and stability as a biological control agent.

It is important to note that in this part of the experiment, larvae were provisioned with dissected snail tissue rather than live, intact prey. Therefore, the measured consumption amount reflects ingestion rate under conditions of easy access to food, not the full predation capacity (which includes search, attack, and handling of live snails). This limitation should be considered when interpreting the ecological relevance of these consumption values.

The study further reveals that *A. leii* larvae are not only behaviorally receptive to *P. canaliculata* as food but also capable of killing it. Predatory efficacy varied significantly among larval instars, with the 4th-instar larvae exhibiting optimal performance, characterized by the shortest mean lethal time, the highest consumption per unit time, and the most prominent snail control efficiency. In contrast, consumption decreased and lethal time increased in later instars (5th–6th), which may be related to their physiological shift towards the prepupal stage and associated changes in energy allocation strategies—a finding consistent with observations in other Coleoptera species [21,23]. This instar-specific pattern provides clear guidance for practical application: when implementing control through mass rearing and release or field population augmentation, priority should be given to utilizing active 3rd and 4th instar larvae to achieve the best cost–benefit ratio and immediate pest suppression effect. Simultaneously, it highlights the need to consider the generation cycle and instar structure in natural population management to ensure sustained control pressure.

This study found that the lethal effect of *A. leii* larvae on *P. canaliculata* is not solely due to physical consumption but is significantly mediated by the injection of digestive tract fluid, with midgut secretions playing a decisive role. The lethal effects of extracts from different parts of *A. leii* varied markedly. No mortality of *P. canaliculata* was observed in the physiological saline control group or the foregut extract treatment group during the observation period, indicating that mere mechanical stimulation or foregut secretions lack significant lethal activity. While mouthpart and hindgut extracts caused mortality in a few individuals, their mortality rates were only 3.3% and 16.7%, respectively, indicating limited efficacy. In stark contrast, the midgut extract achieved a mortality rate as high as 96.7% against *P. canaliculata*, far exceeding that of extracts from other digestive tract sections. This suggests that the midgut likely contains specific enzymes or toxins [18] capable of efficiently disrupting the tissue and physiological functions of *P. canaliculata*. This data further confirms that the digestive fluid or related enzyme systems within the midgut of *A. leii* larvae are the key physiological factors responsible for the death of *P. canaliculata*. It is worth noting that all extracts were standardized to the same concentration (0.5 g tissue equivalent per mL) prior to injection, ensuring that the observed differences in lethal activity reflect intrinsic properties of each digestive tract region rather than concentration variations. The 10 µL dose was selected based on a pre-experiment showing that this volume induced clear mortality without causing immediate death from injection trauma. Future studies should explore dose–response relationships to further characterize the potency of midgut-derived compounds.

Although our experimental design included multiple controls (physiological saline, sterile water, and sham injection) to account for physical injury and solvent effects, we acknowledge that an additional control using gut contents from a non-molluscivorous aquatic insect would further strengthen the specificity of the observed activity. Future studies incorporating such controls, along with investigations into the temporal dynamics of toxin secretion and transport within the digestive tract, will help clarify the mechanisms underlying the potent lethal effect of *A. leii* midgut secretions.

## 5. Conclusions

This study provides the first systematic evidence that larvae of the aquatic firefly *Aquatica leii* have significant potential as a biocontrol agent against the invasive snail *Pomacea canaliculata*. The main findings and their implications are summarized as follows:

*A. leii* larvae readily accept *P. canaliculata* as prey, with the 4th instar exhibiting optimal predatory performance—characterized by the shortest mean lethal time (7.37 min) and the highest weekly consumption (1.23 g). This identifies the 4th instar as the key functional stage for snail suppression and provides practical guidance for mass rearing and release strategies in biocontrol programs.

Midgut crude extract from 4th-instar larvae induced 96.7% mortality in *P. canaliculata* within 12 h, far exceeding the effects of extracts from other digestive tract regions. This suggests that predation by A. leii may involve not only physical consumption but also the action of specific digestive enzymes or toxins, a hypothesis that requires further biochemical validation. Secreted by the midgut, which rapidly incapacitates prey through an “injection–external digestion” mode [18]. This discovery not only explains the high lethal efficiency observed but also points toward the development of novel biogenic molluscicides.

As a native aquatic natural enemy insect [24], *A. leii* possesses clear potential for integration into green control systems for *P. canaliculata*. The dual effectiveness of its predatory behavior and physiological lethal mechanism offers a new pathway to replace or supplement existing chemical methods. Future research should focus on field validation of control efficacy, ecological safety assessment, and isolation and characterization of the midgut active compounds to advance this resource insect from experimental promise to practical application.

## Figures and Tables

**Table 1:** Feeding preference of *Aquatica leii* larvae for *Pomacea canaliculata.*

Observation Metric	Selection of *Pomacea canaliculata* (Individuals)	Selection of *C. chinensis* (Individuals)	Statistical Result
First attack target	13	23	χ^2^ = 2.78, *p* > 0.05
Final feeding target	15	21	χ^2^ = 1.00, *p* > 0.05

**Table 2:** Lethal effect of extracts from different body parts of *Aquatica leii* on *Pomacea canaliculata.*

Injection Fluid	Group 1 Mortality	Group 2 Mortality	Group 3 Mortality	Total Mortality	Mortality (%)
Physiological saline	0/30	0/30	0/30	0/90	0
Mouthpart extract	3/30	0/30	0/30	3/90	3.3
Foregut extract	0/30	0/30	0/30	0/90	0
Midgut extract	27/30	30/30	30/30	87/90	96.7
Hindgut extract	9/30	3/30	3/30	15/90	16.7

Note: Except for the control group, all other injection fluids consisted of a mixture of the respective extract and physiological saline.

## Data Availability

The data that support the findings of this study are openly available.
